# *L*-Methionase: A Therapeutic Enzyme to Treat Malignancies

**DOI:** 10.1155/2014/506287

**Published:** 2014-08-31

**Authors:** Bhupender Sharma, Sukhdev Singh, Shamsher S. Kanwar

**Affiliations:** Department of Biotechnology, Himachal Pradesh University, Summer Hill, Shimla 171 005, India

## Abstract

Cancer is an increasing cause of mortality and morbidity throughout the world. *L*-methionase has potential application against many types of cancers. *L*-Methionase is an intracellular enzyme in bacterial species, an extracellular enzyme in fungi, and absent in mammals. *L*-Methionase producing bacterial strain(s) can be isolated by 5,5′-dithio-*bis*-(2-nitrobenzoic acid) as a screening dye. *L*-Methionine plays an important role in tumour cells. These cells become methionine dependent and eventually follow apoptosis due to methionine limitation in cancer cells. *L*-Methionine also plays an indispensable role in gene activation and inactivation due to hypermethylation and/or hypomethylation. Membrane transporters such as GLUT1 and ion channels like Na^2+^, Ca^2+^, K^+^, and Cl^−^ become overexpressed. Further, the α-subunit of ATP synthase plays a role in cancer cells growth and development by providing them enhanced nutritional requirements. Currently, selenomethionine is also used as a prodrug in cancer therapy along with enzyme methionase that converts prodrug into active toxic chemical(s) that causes death of cancerous cells/tissue. More recently, fusion protein (FP) consisting of *L*-methionase linked to annexin-V has been used in cancer therapy. The fusion proteins have advantage that they have specificity only for cancer cells and do not harm the normal cells.

## 1. Introduction 

*L*-Methionine-*γ*-lyase (EC 4.4.1.11; MGL), also known as methionase, methioninase,* L*-methionine-*γ*-demethiolase, and* L*-methionine methanethiol-lyase (deaminating), is a pyridoxal phosphate (PLP) dependent enzyme. PLP reduces the energy for conversion of amino acids to a zwitterionic carbonion [1] and substantially the apoenzyme catalyzes the cleavage of substrate bond yielding the product [2]. MGL is a cytosolic enzyme inducibly formed by addition of* L*-methionine to the culture medium [3]. MGL has a molecular weight (Mr) of about 149 kDa to 173 kDa and consists of four subunits with identical Mr of about 41 kDa to 45 kDa each except MGL purified to homogeneity from* Pseudomonas putida* (*ovalis*) which was found to consist of two nonidentical subunits of 40 kDa and 48 kDa [4].

**Figure 1:**
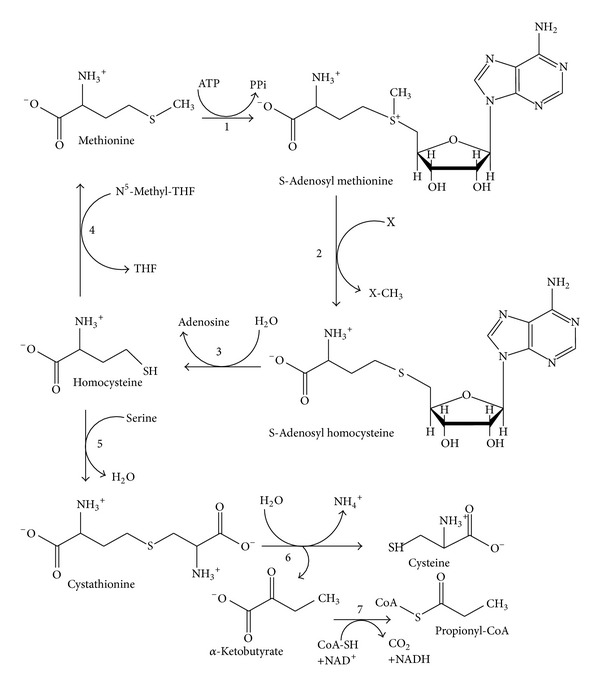
General approach of methionine metabolism (modified from [46]).* L*-Methionine is mainly supplied exogenously from dietary proteins. The enzymes involved in this pathway are (1) methionine adenosyltransferase; (2) S-adenosylmethionine methyltransferases; (3) adenosylhomocysteinase; (4) 5-methyltetrahydrofolate-homocysteine methyltransferase (in mammals betaine-homocysteine methyltransferase or homocysteine methyltransferase); (5) cystathionine-*γ*-synthase; (6) cystathionine-*γ*-lyase; (7) α-ketoacid dehydrogenase.

*L*-Methionine must be incorporated into the human diet in order to biosynthesize* L*-cysteine (Figure 1) by* trans*-sulfuration pathway [5]. In yeast, methionine and cysteine supplementation was required in order to biosynthesize cysteine or methionine, respectively. The microorganisms can synthesize the sulphur containing amino acids by utilizing inorganic sulphate via the* de novo* cysteine biosynthesis pathway [6].* Escherichia coli* and plants utilize the forward* trans*-sulfuration pathway such that methionine is biosynthesized from cysteine or they may utilize inorganic sulphate via* de novo *cysteine biosynthesis [7, 8]. There are different kinds of methionine biosynthesis pathways in different organisms as described in the MetaCyc database.* E. coli* K-12 methionine biosynthesis-I pathway that involves methionine biosynthesis from homoserine, methionine biosynthesis by transsulfuration.* Arabidopsis thaliana*, methionine biosynthesis-II pathway that involves methionine biosynthesis from homoserine-II.* Corynebacterium glutamicum*,* Leptospira meyeri,* and* Saccharomyces cerevisiae* follow methionine biosynthesis-III pathway that performs homoserine methionine biosynthesis and methionine biosynthesis by sufhdrylation.* Arabidopsis thaliana*,* Bacillus subtilis*,* Klebsiella oxytoca*,* Klebsiella pneumonia*,* Lupinus luteus,* and* Oryza sativa* follow methionine salvage-I pathway while* Homo sapiens* and* Rattus norvegicus* possess methionine salvage-II system.* Bacillus subtilis*,* Corynebacterium glutamicum*,* Leptospira meyeri*,* Pseudomonas aeruginosa*,* Pseudomonas putida *and* Saccharomyces cerevisiae* possess a unique superpathway of methionine biosynthesis (by sulfhydrylation). On other hand,* Arabidopsis thaliana*,* Lupinus luteus*,* Oryza sativa*,* Plantago major,* and* Solanum lycopersicum* follow Yang cycle/MTA cycle [9].* E. histolytica *and* T. vaginalis *have a methionine catabolic pathway and elements of a* de novo *sulphide biosynthetic pathway for cysteine biosynthesis in* E. histolytica*. These differences in cysteine metabolism between humans and parasites are of particular interest, especially for the future development of antiparasitic compounds. Currently,* de novo* engineering of a human MGL has been followed for achieving systemic* L*-methionine depletion in cancer therapy [10].

## 2. Sources of MGL

MGL is widely distributed in bacteria, especially in* Pseudomonas* spp. and is induced by the addition of* L*-methionine to the culture medium. Crystal structures of MGL have been reported from* Pseudomonas putida* (*P. putida*) [17, 18],* Citrobacter freundii* [47],* Trichomonas vaginalis* [3], and* Entamoeba histolytica* [21, 22]. The MGL was isolated from different sources such as bacterial, protozoans, fungal, archaeon, and plants (Table 1).

## 3. MGL Isolation 

The bacterial, protozons, archaeal, and plants produce intracellular MGL and fungal sources produce extracellular MGL. Therefore isolation of MGL from microbial sources required cell disruption by chemical, enzymatic, and mechanical methods. Fugal sources produce extracellular MGL; thus, there is no need for cell disruption. Amongst above described MGL sources,* P. putida* is reported to be the best source for MGL production.* P. putida* cell pellets were disrupted by passage through French press. The ammonium sulphate precipitated crude cell lysate was applied on DEAE-cellulose and Sephadex G200 column, respectively. The MGL specific activity (Units/mg protein) from* P. putida* was 14.20 that improved to 3,735 after column chromatography, whereas, in case of* Aspergillus flavipes*, the specific activity (Units/mg protein) was 12.58.* A. flavipes* required 10-day incubation period for growth and 8 days for production time, whereas* P. putida* needed 24–48 h incubation and production time for growth [17, 28]. Microorganisms are most important and convenient sources of commercial enzymes production. Moreover, they have an advantage that they can be cultivated by using inexpensive media and enzyme production occurs in short time.

## 4. Biochemical Reaction Catalyzed by MGL

**Figure 2:**
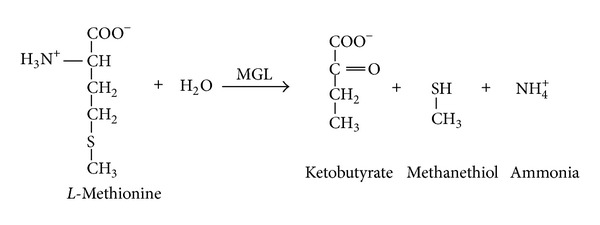
The biochemical reaction catalysed by MGL. MGL catalyses conversion of* L*-methionine into α-ketobutyrate, methanethiol, and ammonia by α, *γ* elimination reaction.

MGL catalyzes the conversion of* L*-methionine to α-ketobutyrate, methanethiol, and ammonia by α, *γ*-elimination reaction (Figure 2).

## 5. Methionase Assay

**Figure 3:**
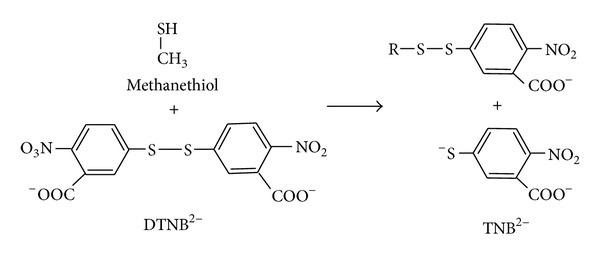
Methionase qualitative detection by DTNB. Methionase catalysis conversion of* L*-methionine as a substrate in agar plates into α-ketobutyrate, ammonia, and methanethiol. DTNB reagent reacts with SH (thiol) functional group and gives intense yellow coloration around methionase producing bacterial isolates.

The free sulphydryl group in solution could be quantitatively measured [48] by 5,5′-dithio-*bis*-(2-nitrobenzoic acid) (DTNB). The DTNB was used as screening dye in agar media to detect methanethiol, which reduces DTNB to form yellow coloured aryl mercaptan (2-nitro-5-thiobenzoate or TNB) around the bacterial colony that is able to produce MGL enzyme. DTNB has little absorbance, but when it reacts with thiol (SH) groups on proteins under mild alkaline conditions (pH 7-8), the 2-nitro-5-thiobenzoate anion (TNB^2-^) gives an intense yellow color (Figure 3). Ellman's reagent is useful assay reagent because of its specificity for SH groups [49] at neutral pH, high molar extinction coefficient, and short reaction time. MGL activity was quantitatively assayed by 3-methyl-2-benzothiazolone hydrazone (MBTH) which determines the amount of α-ketobutyrate produced spectrophotometricalIy at 320 nm. The 3-(4,5-dimethylthiazol-2-yl)-2,5-dimethyl-tetrazolium bromide (MTT) assay [50] was used to determine the* in vitro *growth inhibition of tumour cells by MGL treatment.

## 6. Methionine Requirement in Cancer Cell

**Figure 4:**
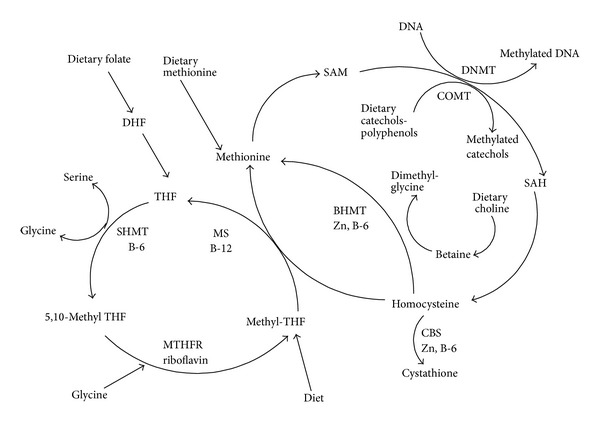
The pathways involved in cellular metabolism and production of S-adenosylmethionine (SAM) for DNA methylation [61]. Dietary factors regulate DNA and histone methylation such as BHMT betaine-homocysteine methyltransferase; CBS cystathionine *β*-synthase; COMT catechol-O-methyltransferase; DHF dihydrofolate; MS methionine synthase; MTHFR 5, 10-methylenetetrahydrofolate reductase; SAH S-adenosyl homocysteine; SAM S-adenosyl methionine; SHMT serine hydroxymethyltransferase and THF tetrahydrofolate.

Tumours cells have uncontrolled rapid growth and proliferation as compared to the normal cells [51]. Many malignant human cell lines have enhanced requirements of methionine for high protein synthesis and regulation of DNA expression in cancer cells [31, 52–57]. Methionine is converted to S-adenosylmethionine and it becomes methyl donor for DNA methylation, an epigenetic phenomenon [58–61] associated with cancer (Figure 4). The high methionine diets were associated with increased prostate cancer risk. The higher availability of* L*-methionine leads to higher bioavailability of S-adenosylmethionine to donate methyl groups to DNA, resulting in DNA hypermethylation of regulatory regions, including tumour suppressors [62, 63].

The CpG is a cytosine-guanosine (CG) dinucleotide DNA sequence, in which the cytosine undergoes chemical modification to contain a methyl group. The methyl binding protein (MBP) primarily was involved in gene regulation of normal cells to exert transcriptional control and also exploited by cancer cells to escape such control [64]. DNA methylation is essential for normal development but in some diseases, such as cancer, gene promoter CpG islands acquire abnormal hypermethylation. The transcriptional silencing due to hypermethylation was inherited by daughter cells following cell division. Alterations of DNA methylation have been recognized to play important role in cancer development. The CpG hypermethylation has been observed in cancer cell lines such as breast, colon, lung, head and neck squamous cell carcinomas, glioblastoma, acute myeloid leukemia, medulloblastoma, and testicular germ cells tumours [65]. UHRF1 (also known as ICBP90) and DNA methyltransferases (DNTs) are involved in maintenance of mammalian DNA methylation. UHRF1 (ubiquitin-like, two zinc-finger domains PHD, RING), also known as NP95 in mouse and ICBP90 in human, is required for maintaining DNA methylation. ICBP90 binds to the methylated retinoblastoma gene (RB1) gene promoter in the G1 phase and allows cells to smoothly enter the S phase [66, 67]. DNMT1 was found to be the sole detectable DNA methyltransferase in all murine tissues and cell types examined till date. Pyrosequencing assays were used to measure the DNA methylation of CDKN2A, RASSF1, CYGB, CDH13, DNMT1, DNMT3A, DNMT3B, and UHRF1 promoters [68]. UHRF1 overexpression in zebrafish hepatocytes causes DNA hypomethylation, Tp53-mediated senescence, and hepatocellular carcinoma [69, 70]. Restriction landmark genomic scanning (RLGS) was also used to assess the methylation in human malignancies.* L*-Methionine downregulates the genes belonging to protein kinase families on MCF-7 breast cancer cells and LNCaP prostate cancer cells and showed antiproliferative effect.* L*-Methionine also activates some of the genes involved in cellular redox regulation [71].* L*-Methionine is required for the biosynthesis of the polyamines spermine and spermidine, which are mainly involved in cell proliferation [72]. The site-specific hypermethylation of cancer-related genes and miRNAs (microRNAs) hypomethylation occur in many cancers. Hypomethylation of miRNAs result in genome instability and activation of protooncogenes. The hypermethylation causes repression of tumour suppressor miRNAs by hypermethylation of their corresponding promoter loci. The miRNAs regulate gene expression within a cell and in the neighboring cells [73–75].

The normal cells have methionine synthase and can form methionine from homocysteine by methyl tetrahydrofolate and betaine as methyl group donors [76]. Methionine-dependent tumour cell lines present no or low levels of methionine synthase [51]. The dependence of tumours on methionine synthase for various cell lines in comparison to the normal cells has been previously reported [77–79].* L*-Methionine is required for the synthesis of vitamins, antioxidants, DNA stabilizers, epigenetic DNA modulators, coenzymes [61, 80, 81], proteins, polyamines (proper cell development), antioxidative stress defense (glutathione/trypanothione), iron-sulfur cluster biosynthesis (energy metabolism), and methylation reactions and it also regulates the gene expression [5–8, 78]. The* L*-methionine is the first amino acid incorporated into many functional proteins during translation and also serves as a precursor for cysteine biosynthesis. Methionine dependence has been observed in many human cancer cell lines and cancer xenografts in animal models [82–84]. Methionine dependence is a metabolic defect seen only in cancer cells and such malignant cells do not grow in a medium in which methionine is depleted [30, 85]. Thus,* L*-methionase has received appreciable attention as a therapeutic agent against various types of methionine dependent tumours [86]. Dietary factors and epigenetic regulator play essential roles in antitumour activities [87]. Several approaches such as starvation of the tumour cells for methionine using methionine-free diets display a reliable efficacy against various types of tumour cells [88]. When tumour cells were deprived of methionine in a homocysteine containing medium* in vitro*, they were reversibly arrested in the late S/G_2_ phase of the cell cycle and finally undergo apoptosis [89–91]. The methionine/valine depleted, tyrosine lowered, and arginine enriched in the diet were the most rationalized form of diet to achieve inhibition of tumour growth [92, 93]. The methionine-free diet is therapeutically not efficient due to economic and technical considerations [88]. A breast cancer cell line MDAMB468 showed methionine dependence and this dependency was due to SAM limitation [94]. There are a few other methionine dependent cell lines (Table 2) reported in the literature.

Cancer cells showed Warburg effect that refers to an increased utilization of glucose via glycolysis and was common in cancerous cells [95]. Glucose transport in cells is rate-limiting step for glucose metabolism mediated by facilitative glucose transporter (GLUT) proteins. The sugar transporters become activated in cancer cells so they incorporate higher amounts of sugar than normal cells. In tumour cells membrane transporter and channel proteins enhance uptake from outer sources and endogenous synthesis increases amongst many transporters glucose transporters (GLUTs) and sodium dependent sugar transporters (SGLT) play main role [96, 97]. The SGLT transporters comprises the sodium-glucose symporter SGLT2 expression was significantly higher in liver and lymph node [98]. The tumour has increased fatty acid synthesis and increased rate(s) of glutamine metabolism. High degree of GLUT1 expression has been reported in human hepatocellular carcinoma, oral cancer [99] and human pancreatic carcinoma (PC) cell line [100–105], and MKN45 (human gastric cancer). The glucose passes through membrane by facilitated diffusion via GLUT or by active transport through a SGLT [106]. Therefore cellular metabolic enzymes such as glucose transporters, hexokinase, pyruvate kinase, lactate dehydrogenase, pyruvate dehydrogenase kinase, fatty acid synthase, and glutaminase targeting enhance the efficacy of common therapeutic agents [95]. GLUT-1 overexpression increased matrix metalloproteinase-2 (MMP-2) promoter activity and was involved in binding of p53 to the MMP-2 promoter [107]. Solute-linked carrier family A1 member 5 (SLC1A5) mediates glutamine transport was overexpressed an associated with squamous lung cancer [108]. CPT-1 transporter helps in fatty acids transport in the form of acyl CoA and converted acetyl CoA. Acetyl CoA enters the TCA cycle and produces NADH which fuels the cell by oxidative phosphorylation [104]. AKT (protein kinase B PKB), a serine/threonine specific protein kinase activation, promotes cell growth, survival, and upregulation of ER-UDP hydrolysis enzyme as observed in human cancers. The ectonucleoside triphosphate diphosphohydrolase 5 (ENTPD5), an endoplasmic reticulum (ER) enzyme, elevated lactate production under aerobic conditions [109]. ENTPD5 expression and AKT activation is common in both cultured prostate cancer cell lines and primary human prostate carcinoma. Lowered ATP/AMP ratio increases glycolysis, elevates lactate production, and provides glycolytic intermediates for biomass production. The overexpressed α-subunit of ATP synthase, in breast cancer, was involved in the progression and metastasis of breast cancer [110, 111]. Periplocin downregulated the ATP synthase ecto-α-subunit (ATP5A1) and eukaryotic translation initiation factor 5A-1 (eIF5A) by periplocin mediated growth inhibition of A549 cells [112]. ATP synthase was upregulated in cancer cells [113, 114]. Ion channels like Na^2+^, Ca^2+^, K^+,^ and Cl^−^ play significant role in cells. The intracellular chloride channel (CLIC) plays an essential role in cellular function, pH, electrogenic balance and maintaining membrane potential in organelles. The chloride channel (CLIC1-5) except CLIC4 became overexpressed in cancer cells. CLIC4 expression reduced in tumour cells [115, 116] and ion channels used to inhibit cancer cell growth [117]. The flow of potassium ions plays important functions, such as cell proliferation, angiogenesis or cell migration, which have also recently been assessed [118, 119]. ABC transporters require energy in the form of adenosine triphosphate (ATP) to translocate substrates across cell membranes. This protein can transport cationic or electrically neutral substrates as well as a broad spectrum of amphiphilic substrates [120]. The ABCG2 (G-subfamily of human ABC) transporter was downregulated in cancer cells [121]. ABC transporters showed multidrug resistance (MDR) in cancer cells by the overexpression of ABC transporters which increased efflux of drugs from cancer cells, thereby decreasing intracellular drug concentration [122, 123].

## 7. Utilization of MGL in Cancer Therapy

### 7.1. Combinational Therapy

**Figure 5:**
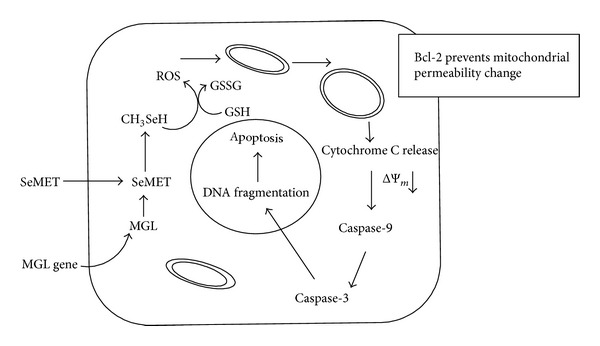
A proposed mechanism of MGL/SeMET-induced apoptosis by combinational therapy method. MGL gene (PpMGL) along with selenomethionine as prodrug was inserted inside the tumour cells. MGL gene product* L*-methionase catalyses the breakdown of methionine from prodrug and synthesizes a toxic molecule “methylselenol” that produces reactive oxygen species (ROS). The ROS thereby causes mitochondrial apoptosis by caspase activation.

Therapeutic exploitation of* P. putida* MGL to deplete plasma methionine has been extensively investigated [65, 66]. The MGL was tested as a potent antiproliferative enzyme towards Lewis lung and human colon carcinoma [124], glioblastoma [33], and neuroblastoma [125]. The cancer cell targeted drugs, that is, small molecules, are not fully effective because cancer stem cells are able to expel the drugs before the cancer cells are destroyed and the cancer cells are then able to renew and produce relapse of the disease. A therapeutic approach to deplete methionine from tumours is to treat the cells with recombinant MGL from* P. putida *[PpMGL]. The growth of human tumours* in vivo *and* in vitro *(xenographed in nude mice) is reported to be inhibited upon treatment with recombinant PpMGL when compared to normal cells [126]. Reduction in cell growth is also achieved by integrating PpMGLgene into human lung cancer cells by using a retroviral-based vector. The treatment with exogenous recombinant PpMGL, in order to deplete intracellular and extracellular methionine (Figure 5) levels, has been attempted [38, 127]. MGL alone or in combination with chemotherapeutic agents such as cisplatin, 5-fluorouracil (5-FU), 1-3-bis(2-chloroethyl)-1-nitrosourea (BCNU), and vincristine has shown efficacy and synergy, respectively, in mouse models of colon cancer, lung cancer, and brain cancer [41, 125, 128]. It was also reported that MGL introduced by adenovirus vector inhibited the growth of tumours* in vitro*. MGL, when combined with selenomethionine [SeMET], a suicide prodrug substrate of MGL, inhibited tumour growth in rodents and prolonged their survivals [127].

The effect of prodrug [Selenomethionine] and the toxic product [Methylselenol] synthesized in the tumour cells is presented below:

(1) Selenomethionine+H2O→L-Methionaseα-Ketobutyrate+Ammonia+Methylselenol

The MGL gene product, α-methionine-*γ*-lyase converts nontoxic SeMET to methylselenol that catalyzes oxidation of thiols to generate toxic superoxide. Apoptosis occurs mainly via a mitochondrial pathway [89]. Methylselenol readily diffused to the surrounding nontransduced tumour cells, destroying the mitochondrial membrane by the oxidative stress [39]. Treatment of the transduced cells with exogenous selenomethionine is found to inhibit tumour cell growth [129]. The methylselenol is required in very low concentration to induce cell cycle arrest and apoptosis [130]. Methylselenol promotes the expression of matrix metalloproteinase (MMP) and tissue inhibitor of metalloproteinase (TIMP) that inhibits the migration of tumour cells [131]. Methylselenol induced apoptosis reported in many cancer cells such as murine melanoma B16F10 [132], fibrosarcoma cells HT1080 [130, 131, 133], colon cancer derived HCT-116 [134], andhuman prostate cancer cells LNCaP [135]. Methylselenol inhibits cell proliferation in the cancerous HCT116 cells as compared to normal cells NCM460 [134]. Methylselenol rapidly decreased cellular prostate-specific antigen (PSA) level in LNCaP cells [135]. ROS promote cell proliferation in low concentration, whereas increase of ROS can induce cell death. Therefore balance between generation and elimination of ROS maintains the proper function. Methylselenol catalyzes the oxidation of thiols, generating toxic ROS causing mitochondrial swelling, releasing cytochrome C, activating the caspase cascade, and inducing the cell apoptosis and death [136]. Selenomethionine is relatively nontoxic to the mammalian cells due to their lack of* L*-methionase. The maximum antiprostate cancers activity was observed by selenomethionine methionase treatment [137]. The sensitivity of tumour cells to selenomethionine was increased by 1,000-fold via transduction by adenovirus mediated methionase gene [138]. The combination of methionase gene, methionase, and selenomethionine are effective against all methionine dependent tumours [39, 127].

### 7.2. Use of Fusion Proteins in Cancer Cell Targeting

The oxidative stress in tumour cells caused exposure of phosphatidylserine on the surface of the vascular endothelium of blood vessels in tumours but not on normal cells [139]. The fusion protein (FP) consisting of* L*-methionase linked to human annexin-V injected into the bloodstream will bind to the marker on vascular endothelial cells of the tumour only. The FP catalyzed the conversion of nontoxic prodrug selenomethionine into toxic methylselenol and also prevented the methionine supplementation to the tumour cells, thereby killing the tumour and/or inhibiting its growth due to methionine restriction [140–142]. The great advantage of FP is that it does not require to be delivered directly to the tumour cells but only to the bloodstream. ATF-methionase FP (amino-terminal fragment of urokinase) was used to inhibit cancer cell proliferation and migration, which supports targeting* L*-methionase to the surface of the cancer cells. The FP has potential as a selective therapeutic agent for the treatment of various methionine-dependent cancers [143].

## 8. Modifications of* L*-Methionase to Reduce Its Side Effects

Tumour growth inhibitory effect of rMGL and PEG-MGL on human cancer cells such as human lung, colon, kidney, brain, prostate, and melanoma cancer cells and lung cancer orthotopic model [38, 144]. It was reported that administration of MGL resulted in a steady-state depletion of plasma methionine to less than 2 *μ*M concentration. The only manifested toxicity was a decreased food intake and a slight weight loss. Serum albumin and red cell values declined transiently during treatment, which might be related to extensive blood sampling, although vomiting was frequently observed in macaque monkeys [145]. To overcome this problem, polyethylene glycol-conjugated MGL (PEG-MGL) was prepared. Simultaneous coadministration of pyridoxal 5′-phosphate and oleic acid or dithiothreitol treatment also strengthened effectiveness of PEG-MGL. To improve the MGL therapeutic potential, MGL was coupled to methoxy polyethylene glycol succinimidyl glutarate-5000 (MEGC-PEG-5000). The half-life due to pegylation increased 6 to 19 times while plasma methionine depletion efficacy decreased 8 to 48 times. Protective effect of high-level of pegylation helps to remove PLP dependence. PEG-rMGL demonstrated a significant decrease in antigenicity [146]. The specific activity of PEG-MGL increased with DTT [147]. Although* L*-methionase from bacterial (prokaryotic) origin has immunogenic issues that can be overcome by PEGylation and by other methods such as deimmunization by combinational T-cell epitope removal using neutral drift [148].

## 9. MGL Cloning

MGL was used for methionine depletion* in vivo* [149]. Bacterial enzymes from various sources have been purified and tested as methionine depleting agents against cancer cell lines. The* P. putida *(pMGL) source was selected for therapeutic applications due to its high catalytic activity, low *K*_*m*_, and a relatively high *k*_cat_ value [17]. The reaction mechanism characterized by using site-directed mutagenesis [150, 151]. The gene(s) for MGL was/were cloned into suitable host cells (Table 3).

## 10. Future Prospective

The unique catalytic reaction of MGL and its limited distribution in pathogens but not in human make this enzyme a promising target to design novel chemotherapeutic agents. Tumour cells show enhanced methionine dependence/requirement in comparison to the normal cells. The greater requirement of methionine by rapidly growing tumour cells supports high protein synthesis and regulation of DNA expression yet it can be exploited by the use of methionase-based therapy to rapidly deplete the cancerous cells. Thus the forced restriction of methionine may be an important strategy in cancer growth control particularly in malignant/cancers that exhibit dependence on methionine for their survival and proliferation. Currently fusion proteins (consisting of* L*-methionase linked to human annexin-V) may have an advantage in comparison to other approaches as they show application in specifically targeting tumour cells without affecting the normal cells.

## Figures and Tables

**Table 1:** Potential sources for *L*-methionine *γ*-lyase isolation.

Source	Examples	Reference(s)
Bacteria	*Brevibacterium linens *	[11]
	*Clostridium sporogenes *	[12]
	*Citrobacter intermedius *	[13]
	*Citrobacter freundii *	[14]
	*Porphyromonas gingivalis *	[15]
	*Pseudomonas ovalis *	[16]
	*Pseudomonas putida *	[17, 18]
	*Treponema denticola *	[19]
	*Micrococcus luteus, Arthrobacter *sp*., Corynebacterium glutamicum *and* Staphylococcus equorum *	[20]

Protozoa	*Trichomonas vaginalis *	[3]
	*Entamoeba histolytica *	[21–23]

Plant	*Arabidopsis thaliana *	[24]

Archaeon	*Ferroplasma acidarmanus *	[25]

Fungus	*Aspergillus* sp. RS-1a	[26]
	*Geotrichum candidum *	[27]
	*Aspergillus flavipes *	[28]
	*Scopulariopsis brevicaulis *	
	*A. carneus *	
	*Penicillium notatum *	
	*Fusarium solani *	

**Table 2:** Cancer cells lines that possess methionine dependency.

Methionine dependent cell line(s)	References
PC-3 cell line human prostate	[29]
Prostate cancer PC3; lung carcinoma SKLU-I; fibrosarcoma HT 1080	[30]
Lung adenocarcinoma A-549 and the acute lymphoblastic leukemia CCRF-HSB-2,	[31]
W 256	[32]
D-54, SWB77 (human glioblastomas) and Daoy (human medulloblastoma)	[33]
Human melanoma cell line MeWoLC1	[34]

**Table 3:** Molecular cloning and functional characterization of MGL gene in various expression systems.

Gene from	Host strain for expression	Selectable marker	References
*P. putida. *	*E. coli* MV1184	Ampicillin	[35]
*P. putida *	*E. coli* BL21 (DE3)	Ampicillin	[36]
*P. putida *ICR3460	*E. coli* JM109	Tetracycline	[37]
*P. putida *	Lung cancer cell line H460	GFP fluorescence, Penicillin, Streptomycin	[38]
*P. putida *	Human lung adenocarcinoma epithelial cell line (A549 cells)	G418 (Geneticin)	[39]
*P. putida* ICR3460	*E. coli* JM109	Tetracycline	[40]
*P. putida* ICR3460	*E. coli* JM109	Tetracycline	[41]
*Trichomonas vaginalis *	*E. coli* M15pREP4	Ampicillin and Kanamycin	[42]
*Trichomonas vaginalis *	*E. coli* M15[pREP4]	Ampicillin and Kanamycin	[43]
*Treponema denticola* ATCC35405	*E. coli* BL21	Ampicillin	[19]
*Entamoeba histolytica *	*E. coli* BL21	Ampicillin	[22]
*Kluyveromyces lactis *CLIB 640	*E. coli *DH10B	Ampicillin	[44]
*Arabidopsis *	*E. coli *BL21	Carbenicillin	[24]
*Brevibacterium linens *	*E. coli *DH5α	Ampicillin	[45]

## References

[1] Richard JP, Amyes TL (2004). On the importance of being zwitterionic: enzymatic catalysis of decarboxylation and deprotonation of cationic carbon. *Bioorganic Chemistry*.

[2] Wolfenden R (2011). Benchmark reaction rates, the stability of biological molecules in water, and the evolution of catalytic power in enzymes. *Annual Review of Biochemistry*.

[3] Lockwood BC, Coombs GH (1991). Purification and characterization of methionine *γ*-lyase from *Trichomonas vaginalis*. *Biochemical Journal*.

[4] Nakayama T, Esaki N, Sugie K, Beresov TT, Tanaka H, Soda K (1984). Purification of bacterial *L*-methionine *γ*-lyase. *Analytical Biochemistry*.

[5] Stipanuk MH (1986). Metabolism of sulfur-containing amino acids.. *Annual review of nutrition*.

[6] Thomas D, Surdin-Kerjan Y (1997). Metabolism of sulfur amino acids in *Saccharomyces cerevisiae*. *Microbiology and Molecular Biology Reviews*.

[7] Ravanel S, Gakière B, Job D, Douce R (1998). The specific features of methionine biosynthesis and metabolism in plants. *Proceedings of the National Academy of Sciences of the United States of America*.

[8] Sekowska A, Kung H, Danchin A (2000). Sulfur metabolism in *Escherichia coli* and related bacteria: facts and fiction. *Journal of Molecular Microbiology and Biotechnology*.

[9] Caspi R, Altman T, Dreher K (2012). The MetaCyc database of metabolic pathways and enzymes and the BioCyc collection of pathway/genome databases. *Nucleic Acids Research*.

[10] Stone E, Paley O, Hu J, Ekerdt B, Cheung N, Georgiou G (2012). De novo engineering of a human cystathionine-*γ*-lyase for systemic *L*-methionine depletion cancer therapy. *ACS Chemical Biology*.

[11] Dias B, Weimer B (1998). Purification and characterization of *L*-methionine *γ*-lyase from *Brevibacterium linens* BL2. *Applied and Environmental Microbiology*.

[12] Kreis W, Hession C (1973). Isolation and purification of *L*-methionine-α-deamino-*γ*-mercapto methane-lyase (*L*-methionase) from *Clostridium sporogenes*. *Cancer Research*.

[13] Faleev NG, Troitskaya MV, Paskonova EA, Saporovskaya MB, Belikov VM (1996). *L*-Methionine-*γ*-lyase in *Citrobacter intermedius* cells: stereochemical requirements with respect to the thiol structure. *Enzyme and Microbial Technology*.

[14] Manukhov IV, Mamaeva DV, Morozova EA (2006). *L*-methionine *γ*-lyase from *Citrobacter freundii*: cloning of the gene and kinetic parameters of the enzyme. *Biochemistry*.

[15] Yoshimura M, Nakano Y, Yamashita Y, Oho T, Saito T, Koga T (2000). Formation of methyl mercaptan from *L*-methionine by *Porphyromonas gingivalis*. *Infection and Immunity*.

[16] Tanaka H, Esaki N, Yamamoto T, Soda K (1976). Purification and properties of methioninase from *Pseudomonas ovalis*. *FEBS Letters*.

[17] Ito S, Nakamura T, Eguchi Y (1976). Purification and characterization of methioninase from *Pseudomonas putida*. *Journal of Biochemistry*.

[18] Kudou D, Misaki S, Yamashita M (2007). Structure of the antitumour enzyme *L*-methionine *γ*-lyase from *Pseudomonas putida* at 1.8 Å resolution. *Journal of Biochemistry*.

[19] Fukamachi H, Nakano Y, Okano S, Shibata Y, Abiko Y, Yamashita Y (2005). High production of methyl mercaptan by *L*-methionine-α-deamino-*γ*-mercaptomethane lyase from *Treponema denticola*. *Biochemical and Biophysical Research Communications*.

[20] Bonnarme P, Psoni L, Spinnler HE (2000). Diversity of *L*-methionine catabolism pathways in cheese-ripening bacteria. *Applied and Environmental Microbiology*.

[21] Sato D, Yamagata W, Kamei K, Nozaki T, Harada S (2006). Expression, purification and crystallization of *L*-methionine *γ*-lyase 2 from *Entamoeba histolytica*. *Acta Crystallographica F: Structural Biology and Crystallization Communications*.

[22] Sato D, Karaki T, Shimizu A, Kamei K, Harada S, Nozaki T (2008). Crystallization and preliminary X-ray analysis of *L*-methionine *γ*-lyase 1 from *Entamoeba histolytica*. *Acta Crystallographica F: Structural Biology and Crystallization Communications*.

[23] Tokoro M, Asai T, Kobayashi S, Takeuchi T, Nozaki T (2003). Identification and characterization of two isoenzymes of methionine-*γ*-lyase from *Entamoeba histolytica*: a key enzyme of sulphur amino acid degradation in an anaerobic parasitic protist that lacks forward and reverse trans-sulfuration pathways. *The Journal of Biological Chemistry*.

[24] Rébeillé F, Jabrin S, Bligny R (2006). Methionine catabolism in *Arabidopsis* cells is initiated by a *γ*-cleavage process and leads to S-methylcysteine and isoleucine syntheses. *Proceedings of the National Academy of Sciences of the United States of America*.

[25] Baumler DJ, Hung K, Jeong KC, Kaspar CW (2007). Production of methanethiol and volatile sulfur compounds by the archaeon *Ferroplasma acidarmanus*. *Extremophiles*.

[26] Ruiz-Herrera J, Starkey RL (1969). Dissimilation of methionine by a demethiolase of *Aspergillus species*. *Journal of Bacteriology*.

[27] Bonnarme P, Lapadatescu C, Yvon M, Spinnler H (2001). *L*-methionine degradation potentialities of cheese-ripening microorganisms. *Journal of Dairy Research*.

[28] Khalaf SA, El-Sayed ASA (2009). *L*-Methioninase production by filamentous fungi: I-screening and optimization under submerged conditions. *Current Microbiology*.

[29] Poirson-Bichat F, Gonfalone G, Bras-Gonçalves RA, Dutrillaux B, Poupon MF (1997). Growth of methionine-dependent human prostate cancer (PC-3) is inhibited by ethionine combined with methionine starvation. *British Journal of Cancer*.

[30] Guo H, Herrera H, Groce A, Hoffman RM (1993). Expression of the biochemical defect of methionine dependence in fresh patient tumors in primary histo-culture. *Cancer Research*.

[31] Kreis W, Goodenow M (1978). Methionine requirement and replacement by homocysteine in tissue cultures of selected rodent and human malignant and normal cells. *Cancer Research*.

[32] Kennelly JC, Blair JA, Pheasant AE (1982). Metabolism of 5-methyltetrahydrofolate by rats bearing the Walker 256 carcinosarcoma. *British Journal of Cancer*.

[33] Kokkinakis DM, Hoffman RM, Frenkel EP (2001). Synergy between methionine stress and chemotherapy in the treatment of brain tumor xenografts in athymic mice. *Cancer Research*.

[34] Watkins D (1998). Cobalamin metabolism in methionine-dependent human tumour and leukemia cell lines. *Clinical and Investigative Medicine*.

[35] Inoue H, Inagaki K, Sugimoto M, Esaki N, Soda K, Tanaka H (1995). Structural analysis of the *L*-methionine *γ*-lyase gene from *Pseudomonas putida*. *The Journal of Biochemistry*.

[36] Tan Y, Xu M, Tan X (1997). Overexpression and large-scale production of recombinant *L*-methionine-α-deamino-*γ*-mercaptomethane-lyase for novel anticancer therapy. *Protein Expression and Purification*.

[37] Takakura T, Ito T, Yagi S (2006). High-level expression and bulk crystallization of recombinant *L*-methionine *γ*-lyase, an anticancer agent. *Applied Microbiology and Biotechnology*.

[38] Miki K, Xu M, An Z (2000). Survival efficacy of the combination of the methioninase gene and methioninase in a lung cancer orthotopic model. *Cancer Gene Therapy*.

[39] Yamamoto N, Gupta A, Xu M (2003). Methioninase gene therapy with selenomethionine induces apoptosis in bcl-2-overproducing lung cancer cells. *Cancer Gene Therapy*.

[40] Takakura T, Mitsushima K, Yagi S (2004). Assay method for antitumor *L*-methionine *γ*- lyase : comprehensive kinetic analysis of the complex reaction with *L* -methionine. *Analytical Biochemistry*.

[41] Yoshioka T, Wada T, Uchida N (1998). Anticancer efficacy *in vivo* and *in vitro*, synergy with 5-fluorouracil, and safety of recombinant methioninase. *Cancer Research*.

[42] McKie AE, Edlind T, Walker J, Mottram JC, Coombs GH (1998). The primitive protozoon *Trichomonas vaginalis* contains two methionine *γ*-lyase genes that encode members of the *γ*-family of pyridoxal 5′-phosphate-dependent enzymes. *The Journal of Biological Chemistry*.

[43] Coombs GH, Mottram JC (2001). Trifluoromethionine, a prodrug designed against methionine *γ*-lyase-containing pathogens, has efficacy *in vitro* and *in vivo* against *Trichomonas vaginalis*. *Antimicrobial Agents and Chemotherapy*.

[44] Kagkli D, Bonnarme P, Neuvéglise C, Cogan TM, Casaregola S (2006). *L*-methionine degradation pathway in *Kluyveromyces lactis*: identification and functional analysis of the genes encoding *L*-methionine aminotransferase. *Applied and Environmental Microbiology*.

[45] Pavani K, Saradhi SV (2014). Cloning and expression of methionine-*γ*-lyase (MGL) of *Brevibacterium linens*. *International Journal of Current Microbiology and Applied Sciences*.

[46] Hori H, Takabayashi K, Orvis L, Carson DA, Nobori T (1996). Gene cloning and characterization of *Pseudomonas putida* L-Methionine-α- deamino-*γ*-mercaptomethane-lyase. *Cancer Research*.

[47] Mamaeva DV, Morozova EA, Nikulin AD (2005). Structure of *Citrobacter freundii* L-methionine *γ*-lyase. *Acta Crystallographica F: Structural Biology and Crystallization Communications*.

[48] Ellman GL (1959). Tissue sulfhydryl groups. *Archives of Biochemistry and Biophysics*.

[49] Habeeb AFSA (1972). Reaction of protein sulphydryl groups with ellmans reagent in enzyme structure. *Methods in Enzymology*.

[50] Alley MC, Scudiero DA, Monks A (1988). Feasibility of drug screening with panels of human tumor cell lines using a microculture tetrazolium assay. *Cancer Research*.

[51] Hoffman RM (1985). Altered methionine metabolism and transmethylation in cancer. *Anticancer Research*.

[52] Halpern BC, Clark BR, Hardy DN, Halpern RM, Smith RA (1974). The effect of replacement of methionine by homocystine on survival of malignant and normal adult mammalian cells in culture. *Proceedings of the National Academy of Sciences of the United States of America*.

[53] Kreis W (1979). Tumor therapy by deprivation of *L*-methionine: rationale and results. *Cancer Treatment Reports*.

[54] Kreis W, Baker A, Ryan V, Bertasso A (1980). Effect of nutritional and enzymatic methionine deprivation upon human normal and malignant cells in tissue culture. *Cancer Research*.

[55] Stern PH, Wallace CD, Hoffman RM (1984). Altered methionine metabolism occurs in all members of a set of diverse human tumor cell lines. *Journal of Cellular Physiology*.

[56] Breillout F, Antoine E, Poupon MF (1990). Methionine dependency of malignant tumors: a possible approach for therapy. *Journal of the National Cancer Institute*.

[57] Swisher EM, Gonzalez RM, Taniguchi T (2009). Methylation and protein expression of DNA repair genes: association with chemotherapy exposure and survival in sporadic ovarian and peritoneal carcinomas. *Molecular Cancer*.

[58] Geiman TM, Muegge K (2010). DNA methylation in early development. *Molecular Reproduction and Development*.

[59] Ramani K, Yang H, Kuhlenkamp J (2010). Changes in the expression of methionine adenosyltransferase genes and S- adenosylmethionine homeostasis during hepatic stellate cell activation. *Hepatology*.

[60] Sen GL, Reuter JA, Webster DE, Zhu L, Khavari PA (2010). DNMT1 maintains progenitor function in self-renewing somatic tissue. *Nature*.

[61] Ho E, Beaver LM, Williams DE, Dashwood RH (2011). Dietary factors and epigenetic regulation for prostate cancer prevention. *Advances in Nutrition*.

[62] Vidal AC, Grant DJ, Williams CD (2012). Associations between intake of folate, methionine, and vitamins B-12, B-6 and prostate cancer risk in American veterans. *Journal of Cancer Epidemiology*.

[63] Das PM, Singal R (2004). DNA methylation and cancer. *Journal of Clinical Oncology*.

[64] Parry L, Clarke AR (2011). The roles of the methyl-CpG binding proteins in cancer. *Genes and Cancer*.

[65] Smiraglia DJ, Rush LJ, Frühwald MC (2001). Excessive CpG island hypermethylation in cancer cell lines versus primary human malignancies. *Human Molecular Genetics*.

[66] Jeanblanc M, Mousli M, Hopfner R (2005). The retinoblastoma gene and its product are targeted by ICBP90: a key mechanism in the G1/S transition during the cell cycle. *Oncogene*.

[67] Bostick M, Jong KK, Estève P, Clark A, Pradhan S, Jacobsen SE (2007). UHRF1 plays a role in maintaining DNA methylation in mammalian cells. *Science*.

[68] Daskalos A, Oleksiewicz U, Filia A (2011). UHRF1-mediated tumor suppressor gene inactivation in nonsmall cell lung cancer. *Cancer*.

[69] Calvisi DF, Simile MM, Ladu S (2007). Altered methionine metabolism and global DNA methylation in liver cancer: relationship with genomic instability and prognosis. *International Journal of Cancer*.

[70] Mudbhary R, Hoshida Y, Chemyavskaya Y (2014). UHRF1 overexpression drives DNA hypomethylation and hepatocellular carcinoma. *Cancer Cell*.

[71] Benavides MA, Hu D, Baraoidan MK (2011). *L*-methionine-induced alterations in molecular signatures in MCF-7 and LNCaP cancer cells. *Journal of Cancer Research and Clinical Oncology*.

[72] Thomas T, Thomas TJ (2001). Polyamines in cell growth and cell death: molecular mechanisms and therapeutic applications. *Cellular and Molecular Life Sciences*.

[73] Lujambio A, Ropero S, Ballestar E (2007). Genetic unmasking of an epigenetically silenced microRNA in human cancer cells. *Cancer Research*.

[74] Mathonnet G, Fabian MR, Svitkin YV (2007). MicroRNA inhibition of translation initiation *in vitro* by targeting the cap-binding complex eIF4F. *Science*.

[75] Lopez-Serra P, Esteller M (2012). DNA methylation-associated silencing of tumor-suppressor microRNAs in cancer. *Oncogene*.

[76] Kenyon SH, Waterfield CJ, Timbrell JA, Nicolaou A (2002). Methionine synthase activity and sulphur amino acid levels in the rat liver tumour cells HTC and Phi-1. *Biochemical Pharmacology*.

[77] Liteplo RG, Hipwell SE, Rosenblatt DS, Sillaots S, Lue-Shing H (1991). Changes in cobalamin metabolism are associated with the altered methionine auxotrophy of highly growth autonomous human melanoma cells. *Journal of Cellular Physiology*.

[78] Davis CD, Uthus EO (2004). DNA methylation, cancer susceptibility, and nutrient interactions. *Experimental Biology and Medicine*.

[79] El-Sayed ASA (2010). Microbial *L*-methioninase, molecular characterization and therapeutic applications. *Applied Microbiology and Biotechnology*.

[80] Cellarier E, Durando X, Vasson MP (2003). Methionine dependency and cancer treatment. *Cancer Treatment Reviews*.

[81] Thivat E, Durando X, Demidem A (2007). A methionine-free diet associated with nitrosourea treatment down-regulates methylguanine-DNA methyl transferase activity in patients with metastatic cancer. *Anticancer Research*.

[82] Fiskerstrand T, Christensen B, Tysnes OB, Ueland PM, Refsum H (1994). Development and reversion of methionine dependence in a human glioma cell line: relation to homocysteine remethylation and cobalamin status. *Cancer Research*.

[83] Hoffman RM, Erbe RW (1976). High in vivo rates of methionine biosynthesis in transformed human and malignant rat cells auxotrophic for methionine. *Proceedings of the National Academy of Sciences of the United States of America*.

[84] Mecham JO, Rowitch D, Wallace CD, Stern PH, Hoffman RM (1983). The metabolic defect of methionine dependence occurs frequently in human tumor cell lines. *Biochemical and Biophysical Research Communications*.

[85] Hoffman RM (1983). Altered methionine metabolism, DNA methylation and oncogene expression in carcinogenesis: a review and synthesis. *Biochimica et Biophysica Acta*.

[86] Kokkinakis DM, Schold SC, Hori H, Nobori T (1997). Effect of long-term depletion of plasma methionine on the growth and survival of human brain tumor xenografts in athymic mice. *Nutrition and Cancer*.

[87] Gerhäuser C (2012). Cancer cell metabolism, epigenetics and the potential influence of dietary components—a perspective. *Biomedical Research*.

[88] Goseki N, Yamazaki S, Endo M (1992). Antitumor effect of methionine-depleting total parenteral nutrition with doxorubicin administration on Yoshida sarcoma-bearing rats. *Cancer*.

[89] Guo H, Lishko VK, Herrera H, Groce A, Kubota T, Hoffman RM (1993). Therapeutic tumor-specific cell cycle block induced by methionine starvation *in vivo*. *Cancer Research*.

[90] Hoffman RM, Jacobsen SJ (1980). Reversible growth arrest in simian virus 40-transformed human fibroblasts. *Proceedings of the National Academy of Sciences of the United States of America*.

[91] Nagahama T, Goseki N, Endo M (1998). Doxorubicin and vincristine with methionine depletion contributed to survival in the Yoshida sarcoma bearing rats. *Anticancer Research*.

[92] He YC, Wang YH, Cao J, Chen JW, Pan DY, Zhou YK (2003). Effect of complex amino acid imbalance on growth of tumor in tumor-bearing rats. *World Journal of Gastroenterology*.

[93] He Y, Cao J, Chen J, Pan D, Zhou Y (2003). Influence of methionine/valine-depleted enteral nutrition on nucleic acid and protein metabolism in tumor-bearing rats. *World Journal of Gastroenterology*.

[94] Booher K, Lin D, Borrego SL, Kaiser P (2012). Downregulation of Cdc6 and pre-replication complexes in response to methionine stress in breast cancer cells. *Cell Cycle*.

[95] Zhao Y, Butler EB, Tan M (2013). Targeting cellular metabolism to improve cancer therapeutics. *Cell Death and Disease*.

[96] Aparicio LA, Calvo MB, Figueroa A, Pulido EG, Campelo RG (2010). Potential role of sugar transporters in cancer and their relationship with anticancer therapy. *International Journal of Endocrinology*.

[97] Diaz-Ruiz R, Rigoulet M, Devin A (2011). The Warburg and Crabtree effects: On the origin of cancer cell energy metabolism and of yeast glucose repression. *Biochimica et Biophysica Acta*.

[98] Ishikawa N, Oguri T, Isobe T, Fujitaka K, Kohno N (2001). SGLT gene expression in primary lung cancers and their metastatic lesions. *Japanese Journal of Cancer Research*.

[99] Oliver RJ, Woodwards RTM, Sloan P, Thakker NS, Stratford IJ, Airley RE (2004). Prognostic value of facilitative glucose transporter Glut-1 in oral squamous cell carcinomas treated by surgical resection: results of EORTC Translational Research Fund studies. *European Journal of Cancer*.

[100] Younes M, Lechago LV, Somoano JR, Mosharaf M, Lechago J (1996). Wide expression of the human erythrocyte glucose transporter Glut1 in human cancers. *Cancer Research*.

[101] Reske SN, Grillenberger KG, Glatting G (1997). Overexpression of glucose transporter 1 and increased FDG uptake in pancreatic carcinoma. *Journal of Nuclear Medicine*.

[102] Ito T, Noguchi Y, Satoh S, Hayashi H, Inayama Y, Kitamura H (1998). Expression of facilitative glucose transporter isoforms in lung carcinomas: its relation to histologic type, differentiation grade and tumor stage. *Modern Pathology*.

[103] Noguchia Y, Saitoa A, Miyagib Y (2000). Suppression of facilitative glucose transporter-1 mRNA can suppress tumor growth. *Cancer Letters*.

[104] Macheda ML, Rogers S, Best JD (2005). Molecular and cellular regulation of glucose transporter (GLUT) proteins in cancer. *Journal of Cellular Physiology*.

[105] Pizzi S, Porzionato A, Pasquali C (2009). Glucose transporter-1 expression and prognostic significance in pancreatic carcinogenesis. *Histology and Histopathology*.

[106] McCracken AN, Edinger AL (2013). Nutrient transporters: the Achilles' heel of anabolism. *Trends in Endocrinology and Metabolism*.

[107] Ito S, Fukusato T, Nemoto T, Sekihara H, Seyama Y, Kubota S (2002). Coexpression of glucose transporter 1 and matrix metalloproteinase-2 in human cancers. *Journal of the National Cancer Institute*.

[108] Hassanein M, Hoeksema MD, Shiota M (2013). SLC1A5 mediates glutamine transport required for lung cancer cell growth and survival. *Clinical Cancer Research*.

[109] Fang M, Shen Z, Huang S (2010). The ER UDPase ENTPD5 promotes protein N-glycosylation, the Warburg effect, and proliferation in the PTEN pathway. *Cell*.

[110] Huang TC, Chang HY, Hsu CH, Kuo WH, Chang KJ, Juan HF (2008). Targeting therapy for breast carcinoma by ATP synthase inhibitor aurovertin B. *Journal of Proteome Research*.

[111] Pan J, Sun L, Tao Y (2011). ATP synthase ecto-α-subunit: a novel therapeutic target for breast cancer. *Journal of Translational Medicine*.

[112] Lu Z, Song Q, Yang J (2014). Comparative proteomic analysis of anti-cancer mechanism by periplocin treatment in lung cancer cells. *Cellular Physiology and Biochemistry*.

[113] Israelsen WJ, Heiden MGV (2010). ATP consumption promotes cancer metabolism. *Cell*.

[114] Ma Z, Cao M, Liu Y (2010). Mitochondrial F1Fo-ATP synthase translocates to cell surface in hepatocytes and has high activity in tumor-like acidic and hypoxic environment. *Acta Biochimica et Biophysica Sinica*.

[115] Suh KS, Mutoh M, Gerdes M, Yuspa SH (2005). CLIC4, an intracellular chloride channel protein, is a novel molecular target for cancer therapy. *Journal of Investigative Dermatology Symposium Proceedings*.

[116] Suh KS, Crutchley JM, Koochek A (2007). Reciprocal modifications of CLIC4 in tumor epithelium and stroma mark malignant progression of multiple human cancers. *Clinical Cancer Research*.

[117] Arcangeli A, Crociani O, Lastraioli E, Masi A, Pillozzi S, Becchetti A (2009). Targeting ion channels in cancer: a novel frontier in antineoplastic therapy. *Current Medicinal Chemistry*.

[118] Pardo LA, Stuhmer W (2014). The roles of K^+^ channels in cancer. *Nature Reviews Cancer*.

[119] Urrego D, Tomczak AP, Zahed F, Stuhmer W, Pardo LA (2014). Potassium channels in cell cycle and cell proliferation. *Philosophical Transactions of the Royal Society*.

[120] Pohl A, Devaux PF, Herrmann A (2005). Function of prokaryotic and eukaryotic ABC proteins in lipid transport. *Biochimica et Biophysica Acta*.

[121] Ishikawa T, Nakagawa H, Hagiya Y, Nonoguchi N, Miyatake S, Kuroiwa T (2010). Key role of human ABC transporter ABCG2 in photodynamic therapy and photodynamic diagnosis. *Advances in Pharmacological Sciences*.

[122] Hlavata I, Mohelnikova-Duchonova B, Vaclavikova R (2012). The role of ABC transporters in progression and clinical outcome of colorectal cancer. *Mutagenesis*.

[123] Sun Y, Patel A, Kumar P, Chen Z (2012). Role of ABC transporters in cancer chemotherapy. *Chinese Journal of Cancer*.

[124] Tan Y, Sun X, Xu M (1998). Polyethylene glycol conjugation of recombinant methioninase for cancer therapy. *Protein Expression and Purification*.

[125] Hu J, Cheung NV (2009). Methionine depletion with recombinant methioninase: In vitro and in vivo efficacy against neuroblastoma and its synergism with chemotherapeutic drugs. *International Journal of Cancer*.

[126] Tan Y, Zavala J, Han Q (1997). Recombinant methioninase infusion reduces the biochemical endpoint of serum methionine with minimal toxicity in high-stage cancer patients. *Anticancer Research*.

[127] Miki K, Xu M, Gupta A (2001). Methioninase cancer gene therapy with selenomethionine as suicide prodrug substrate. *Cancer Research*.

[128] Tan Y, Sun X, Xu M (1999). Efficacy of recombinant methioninase in combination with cisplatin on human colon tumors in nude mice. *Clinical Cancer Research*.

[129] Zhao R, Domann FE, Zhong W (2006). Apoptosis induced by selenomethionine and methioninase is superoxide mediated and p53 dependent in human prostate cancer cells. *Molecular Cancer Therapeutics*.

[130] Zeng H, Wu M, Botnen JH (2009). Methylselenol, a selenium metabolite, induces cell cycle arrest in G1 phase and apoptosis via the extracellular-regulated kinase 1/2 pathway and other cancer signaling genes. *Journal of Nutrition*.

[131] Zu K, Bihani T, Lin A, Park Y-, Mori K, Ip C (2006). Enhanced selenium effect on growth arrest by BiP/GRP78 knockdown in p53-null human prostate cancer cells. *Oncogene*.

[132] Kim A, Oh J, Park J, Chung A (2007). Methylselenol generated from selenomethionine by methioninase downregulates integrin expression and induces caspase-mediated apoptosis of B16F10 melanoma cells. *Journal of Cellular Physiology*.

[133] Zeng H, Briske-Anderson M, Idso JP, Hunt CD (2006). The selenium metabolite methylselenol inhibits the migration and invasion potential of HT1080 tumor cells. *Journal of Nutrition*.

[134] Zeng H, Briske-Anderson M, Wu M, Moyer MP (2012). Methylselenol, a selenium metabolite, plays common and different roles in cancerous colon HCT116 cell and noncancerous NCM460 colon cell proliferation. *Nutrition and Cancer*.

[135] Cho SD, Jiang C, Malewicz B (2004). Methyl selenium metabolites decrease prostate-specific antigen expression by inducing protein degradation and suppressing androgen-stimulated transcription. *Molecular Cancer Therapeutics*.

[136] Green DR, Reed JC (1998). Mitochondria and apoptosis. *Science*.

[137] Li P, Nijhawan D, Budihardjo I (1997). Cytochrome c and dATP-dependent formation of Apaf-1/caspase-9 complex initiates an apoptotic protease cascade. *Cell*.

[138] Miki K, Al-Refaie W, Xu M (2000). Methioninase gene therapy of human cancer cells is synergistic with recombinant methioninase treatment. *Cancer Research*.

[139] Stafford JH, Thorpe PE (2011). Increased exposure of phosphatidylethanolamine on the surface of tumor vascular endothelium. *Neoplasia*.

[140] Harrison RG, Norman Enzyme prodrug cancer therapy selectively targeted to tumor cells or tumor vasculature and methods of production and use thereof.

[141] Rite BDV, Krais JJ, Cherry M, Sikavitsas VI, Kurkjian C, Harrison RG (2013). Antitumor activity of an enzyme prodrug therapy targeted to the breast tumor vasculature. *Cancer Investigation*.

[142] van Rite BD, Lazrak YA, Pagnon ML (2011). Enzyme prodrug therapy designed to target *L*-methioninase to the tumor vasculature. *Cancer Letters*.

[143] Palwai NR, Zang X, Harrison RG, Benbrook D, Pento JT (2009). Selective growth inhibition of cancer cells by L-methioninase-containing fusion protein targeted to the urokinase receptor. *Pharmacology*.

[144] Tan Y, Xu M, Hoffman RM (2010). Broad selective efficacy of recombinant methioninase and polyethylene glycol-modified recombinant methioninase on cancer cells *in vitro*. *Anticancer Research*.

[145] Yang Z, Wang J, Yoshioka T (2004). Pharmacokinetics, methionine depletion and antigenicity of recombinant methioninase in primates. *Clinical Cancer Research*.

[146] Sun X, Yang Z, Li S (2003). *In vivo* efficacy of recombinant methioninase is enhanced by the combination of polyethylene glycol conjugation and pyridoxal 5′-phosphate supplementation. *Cancer Research*.

[147] Takakura T, Takimoto A, Notsu Y (2006). Physicochemical and pharmacokinetic characterization of highly potent recombinant *L*-methionine *γ*-lyase conjugated with polyethylene glycol as an antitumor agent. *Cancer Research*.

[148] Cantor JR, Yoo TH, Dixit A, Iverson BL, Forsthuber TG, Georgiou G (2011). Therapeutic enzyme deimmunization by combinatorial T-cell epitope removal using neutral drift. *Proceedings of the National Academy of Sciences of the United States of America*.

[149] Lishko VK, Lishko OV, Hoffman RM (1993). Depletion of serum methionine by methioninase in mice. *Anticancer Research*.

[150] Inoue H, Inagaki K, Adachi N (2000). Role of tyrosine 114 of L-methionine *γ*-lyase from *Pseudomonas putida*. *Bioscience, Biotechnology and Biochemistry*.

[151] Kudou D, Misaki S, Yamashita M, Tamura T, Esaki N, Inagaki K (2008). The role of cysteine 116 in the active site of the antitumor enzyme *L*-methionine *γ*-lyase from *Pseudomonas putida*. *Bioscience, Biotechnology and Biochemistry*.

